# Vital Statistics of the Census

**Published:** 1902-03

**Authors:** 


### Vital Statistics of the Census.

WE ARE in receipt of an important communication from the United States Census Office, inviting the JOURNAL to coöperate with that office and with the American Public Health Association in an effort to improve and to make uniform over our entire country the nomenclature and the practice of reporting and recording deaths,—an important portion of vital statistics. This movement is wise and timely; upon this question we have decided convictions and we urge its importance with all emphasis.

The facts in the case constitute a scandal of such proportions and nature as should stir to action every member of the medical profession, and should cause such members of the American

Medical Association as still have the ability and inclination to think upon such problems to reflect that possibly it might be better for a time to stop disputing over ethical distinctions, and directions as to hand-washing and hair-parting, and to devote their spare time to more ennobling efforts.

The 12th United States Census gives the mortality statistics for the year 1900, and deems it of importance to state that "the registration area has increased almost 50 per cent. over 1890, and now comprehends nearly 29,000,000 population." This registration area is so named because within this area provision is made for the intelligent registration of deaths, in such form and manner that the data is fit for use in the preparation of the national census.

At the time of the last census this registration area included the District of Columbia and the States of Connecticut, Delaware, Maine, Massachusetts, Michigan, New Hampshire, New Jersey, New York, Rhode Island, and Vermont.

There is also a nonregistration area and this included more than 47,000,000 population—and in this vast area no provision is made for the registration of deaths in such form and manner as is fit for use in the making of a census, and this nonregistration area comprehends the balance of the country including with others such states as Illinois, Indiana, Iowa, Ohio and Pennyslvania. It seems incredible that our advance in civilization is so superficial and unreal that so large a part of our country should be subject to such criticism.

We need, at once, and for all, a uniform nomenclature and a uniform and universal method of registration of deaths, and we call upon the medical men of the several states where this does not exist to cease not from fasting, from prayer, and from agitation, until the desired end be accomplished.

Of course local laws must have local provisions but the general provisions recommended by the census office are so few, simple, and obviously essential that it should be easy to include them. The suggestions are that provision be made (1) that deaths be registered immediately after their occurrence; (2) that certificates of death should be required; (3) that burial or removal permits are essential to the enforcement of the law; (4) that efficient local registrars are necessary; (5) that the responsibility for reporting deaths to the local registrar should be fixed; (6) that the central registration officer should have full control of the local machinery and its rules should have the effect of law; (7) that the transmission and preservation of records should be provided for; (8) that penalties for neglect or errors should be exacted.

How this scandal makes plain the weakness of the medical profession in the several states as a factor of progress and how plain it is that medical schism and dissension has much to answer for as a cause of medical inefficiency or impotency in public matters!

We venture to prophesy that in a decade the united medical profession of the United States could place in the cabinet a Secretary of Health, who should rank next after the Secretary of War, and that in the following decade this officer, properly administering his trust and adequately supported by the same united profession, could so increase the general efficiency of that profession as to cut the death-rate more than 50 per cent.; could increase the average expectation of life of the American citizen by at least ten years, and that in doing this they would only bring the practice of medicine up to the level of its science.

---

THE relation of smallpox to swine has been investigated by the department of health of Buffalo, and has been reported upon by the bacteriologist and local inspector of the bureau of animal industry. We quote the following from the report:

As regards results and final conclusions, although the work is far from completion at the present time, the following can be stated:

1. That the unusual disease noticed in swine is not of the same nature as smallpox.

2. That in the opinion of investigators, it is impossible by any known means of inoculation, to communicate smallpox to swine.

3. That there is an infectious agent present in vaccine that is distinct from that present in the virus of smallpox.

4. That the peculiar dermal disturbance, as seen in the swine is of unusual occurrence in this section of the country, and so far as it has been possible for us to ascertain it is not described in the literature of the day.

5. That inoculation of swine with the virus of smallpox does not confer immunity to vaccinia.

6. That most of the reports of the transmission of smallpox to the lower animals are without scientific substantiation.

7. That the investigators would suggest that with a more complete study into the nature of the variolæ of the different domesticated animals, a greater distinction, rather than a closer relationship, will be established, and that the relationship between these diseases and smallpox, with a possible few exceptions, will be found equally remote.

By a law of Connecticut narcotic and alcoholic patients may voluntarily commit themselves to a sanitarium in that state for treatment for any length of time not exceeding one year. Chapter 230, Section 3690, General Statutes of Connecticut, reads as follows:

The managers, trustees or directors of any inebriate asylum established by the laws of this state, may receive any inebriate or dipsomaniac who shall apply and be received into such an asylum, retain him one year and treat and restrain him in the same manner as if committed by the Probate Court.

